# Post-mortem diagnosis of CACT deficiency with a novel *SLC25A20* variant

**DOI:** 10.1038/s41439-026-00354-2

**Published:** 2026-06-12

**Authors:** Hiromi Nyuzuki, Aya Miura, Takuma Yamamoto, Akihide Koyama, Kazuhisa Funayama, Satoru Takami, Saori Fujita, Youko Kuriyama, Hisakazu Takatsuka, Hajime Nishio, Takeshi Ikeuchi

**Affiliations:** 1 Center for Medical Genetics, Niigata University Medical and Dental Hospital, Niigata, Japan; 2 Department of Pediatrics, Niigata University School of Medicine, Niigata, Japan; 3 Department of Legal Medicine, Hyogo Medical University, Hyogo, Japan; 4 Division of Forensic Medicine, Niigata University Graduate School of Medicine, Dentistry and Health Sciences, Niigata, Japan; 5 Center of Cause of Death Investigation, Niigata University Graduate School of Medicine, Dentistry and Health Sciences, Niigata, Japan; 6 Department of Pediatrics, Saiseikai Niigata Hospital, Niigata, Japan

**Keywords:** Medical genetics, Clinical genetics

## Abstract

Carnitine-acylcarnitine translocase deficiency is a severe neonatal metabolic disorder caused by *SLC25A20* variants. We report a case of sudden neonatal death in which post-mortem CT revealed diffuse fatty liver, and subsequent genetic analysis identified compound heterozygous variants in *SLC25A20*, including a known variant, c.824G>A p.(Arg275Gln), and a novel nonsense variant, c.334C>T p.(Gln112Ter). These findings demonstrate the utility of post-mortem genetic analysis in determining the cause of sudden neonatal death.

Carnitine-acylcarnitine translocase (CACT) deficiency (OMIM 212138) is a rare autosomal recessive disorder of mitochondrial long-chain fatty acid β-oxidation caused by pathogenic variants in *SLC25A20* (refs. ^1,2^). CACT is a key component of the carnitine shuttle system, mediating the exchange of long-chain acylcarnitines and free carnitine across the inner mitochondrial membrane, thereby enabling mitochondrial β-oxidation of long-chain fatty acids^3^. Disruption of this transport process leads to impaired energy production, particularly during fasting or catabolic stress, and results in accumulation of long-chain acylcarnitines and secondary metabolic disturbances^4^. These metabolic abnormalities are known to predominantly affect tissues with high energy demand, including the heart, liver, and skeletal muscle.

Clinically, CACT deficiency most commonly presents in the neonatal period and is characterized by hypoketotic hypoglycemia, hyperammonemia, cardiomyopathy, arrhythmia, hepatomegaly, and rapid progression to multiorgan failure^5,6^. The neonatal form is typically severe and frequently results in death within the first few days of life^6^. Although milder phenotypes and later-onset cases have been reported, these are uncommon, and the clinical spectrum has been described in a limited number of patients^5,6^. Early fatal neonatal cases remain particularly challenging to diagnose, as clinical deterioration often occurs before metabolic evaluation can be performed. In such situations, post-mortem investigations, including genetic analysis, may provide critical information for establishing a definitive diagnosis, particularly in cases of sudden neonatal death.

The patient was the first child of non-consanguineous healthy Japanese parents. There was no family history of sudden infant death. Prenatal ultrasonography revealed no structural abnormalities. She was born at 40 weeks of gestation by normal vaginal delivery, with a birth weight of 2,580 g (−1.31 standard deviation (SD)), length of 46.6 cm (−1.53 SD), and head circumference of 32.5 cm (−0.69 SD). Apgar scores were 9 and 10 at 1 min and 5 min, respectively.

At 1 day of age, her body weight was 2492 g, and no abnormal findings were observed. She was breastfed at ~2–5-h intervals. At ~30 h after birth and 1 h after the last feeding, she was found to be apneic. The infant had been confirmed alive ~5 min before being found unresponsive. Cardiac arrest was recognized, and resuscitation was immediately initiated with endotracheal intubation, bag-valve ventilation, and chest compressions. Epinephrine was administered three times via the endotracheal tube; however, return of spontaneous circulation was not achieved. Resuscitation efforts were continued for more than 1 h without response, and the patient died at 2 days of age. Post-mortem CT, performed with parental consent, demonstrated diffuse fatty liver (Fig. 1a) without other significant abnormalities, whereas conventional autopsy was declined.

**Fig. 1:**
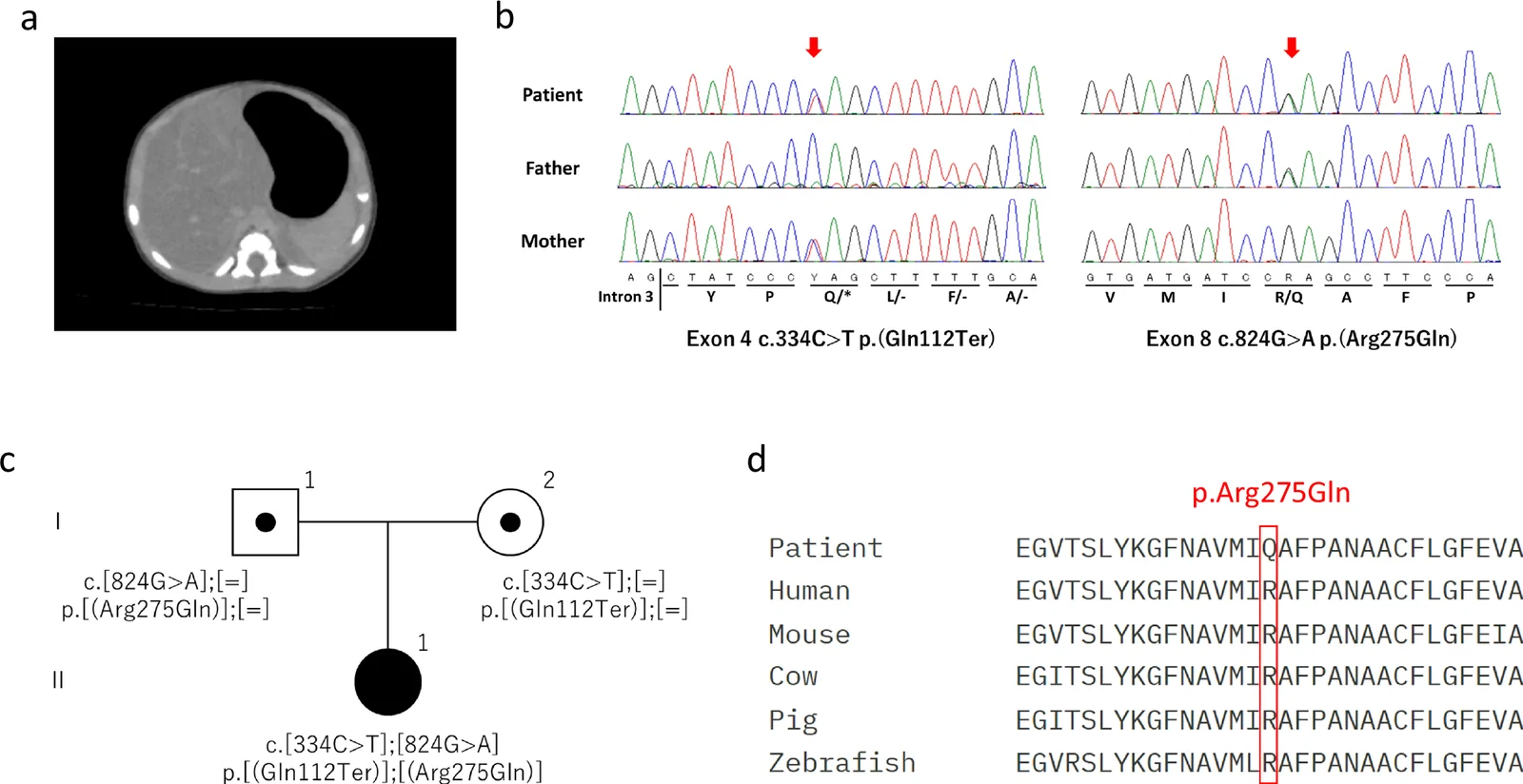
Radiological features and genetic analysis. **a** Post-mortem CT image. Diffuse fatty liver was observed. **b** Sanger sequencing of the patient and the parents. **c** Family pedigree of the patient. **d** Multiple sequence alignment of *SLC25A20* across representative species showing conservation of the Arg275 residue.

To investigate the underlying genetic cause, clinical exome sequencing (TruSight One sequencing panel; Illumina, San Diego, CA, USA) of the patient’s parents was performed on Illumina MiSeq (Illumina), as previously described^7^. Given that only a dried umbilical cord specimen was available from the patient, clinical exome sequencing was performed only in the parents using DNA extracted from peripheral blood. A list of TruSight One genes is available online at http://products.illumina.com/products/trusight-one-sequencing-panel.html. On average, approximately 15 million reads were produced and approximately 9 million reads were mapped to the target regions. The mean coverage of the coding sequences (father/mother) was 73.5/76.7 reads, with an overall average gene-level coverage of >20 reads at 91.5%/92.5%. There were 7,696/7,946 single-nucleotide variants, 168/184 insertions, and 221/228 deletions detected. Synonymous variants without amino acid changes were excluded from further analysis. After filtering as previously described^8^, the total number of detected variants was 483/576.

The presence of fatty liver suggested an underlying inherited metabolic disorder, and variant filtering was therefore focussed on genes associated with inherited metabolic diseases. This analysis identified heterozygous variants in *SLC25A20* (NM_000387.6):c.824G>A p.(Arg275Gln) in the father and a novel c.334C>T p.(Gln112Ter) in the mother. Subsequent Sanger sequencing of DNA extracted from a dried umbilical cord specimen confirmed that the patient harbored these variants in a compound heterozygous state (Fig. 1b, c).

In the Genome Aggregation Database (gnomAD v4.1.1), the allele frequency of c.824G>A p.(Arg275Gln) is 0.000006815 in the total population and 0.00004454 in East Asians, whereas c.334C>T p.(Gln112Ter) was not identified. In ClinVar, c.824G>A p.(Arg275Gln) is classified as pathogenic/likely pathogenic, whereas c.334C>T p.(Gln112Ter) has not been registered. For the missense variant c.824G>A p.(Arg275Gln), multiple in silico prediction tools suggested a deleterious effect on protein function (SIFT score: 0.00; MutationTaster: 1; CADD PHRED score: 34), and the affected residue is evolutionarily conserved across multiple species (Fig. 1d). According to ACMG/AMP guidelines^9^, the c.334C>T p.(Gln112Ter) variant is classified as pathogenic (PVS1, PM2, and PM3), whereas the c.824G>A p.(Arg275Gln) variant is classified as likely pathogenic (PS1, PM1, PM2, and PP3). The identification of these variants in trans provides strong molecular evidence supporting the diagnosis of CACT deficiency.

In this study, we report a Japanese female neonate diagnosed with CACT deficiency by post-mortem genetic analysis. Although biochemical confirmation was not available in this case, the clinical presentation and post-mortem CT findings were consistent with severe neonatal-onset CACT deficiency. The abrupt onset of symptoms shortly after a feeding interval suggests impaired utilization of fatty acids as an alternative energy source during a brief fasting period, which is a characteristic feature of fatty acid oxidation disorders. The presence of diffuse fatty liver further supports impaired mitochondrial β-oxidation and accumulation of lipid substrates in hepatocytes^10^.

Diagnosis of CACT deficiency in early fatal neonatal cases remains challenging. Many patients develop symptoms before the results of newborn screening are available, limiting opportunities for biochemical diagnosis and early intervention^6^. Previous studies in Japan have similarly shown that neonatal-onset CACT deficiency often presents within the first few days of life and may lead to rapid clinical deterioration before newborn screening results become available^11–13^. Furthermore, the acylcarnitine profile of CACT deficiency overlaps with that of carnitine palmitoyltransferase II deficiency, complicating biochemical differentiation^2^. These diagnostic limitations highlight the importance of genetic testing in establishing a definitive diagnosis.

Post-mortem genetic analysis, including metabolic autopsy, has emerged as a valuable approach for determining the cause of sudden unexplained death, particularly in infants^14–16^. In inherited metabolic disorders, in which biochemical data may be unavailable or inconclusive, genetic analysis can provide critical diagnostic information. In the present case, post-mortem genetic testing enabled identification of the underlying cause of death, despite the absence of autopsy and biochemical investigations.

The c.824G>A p.(Arg275Gln) variant has previously been reported in Japanese patients with CACT deficiency and is considered a recurrent allele in this population^12^. The Arg275 residue is located within a functionally important region of the protein, as indicated by prior structural and functional analyses of *SLC25A20*, and shows strong evolutionary conservation across species^17^. By contrast, the c.334C>T p.(Gln112Ter) variant identified in this case represents a novel nonsense variant in *SLC25A20*. Genotype–phenotype correlations in CACT deficiency remain incompletely understood due to the rarity of the condition^5,6^. However, previous reports suggest that truncating variants and missense variants affecting highly conserved residues are associated with more severe phenotypes^5,18^. The combination of a predicted loss-of-function variant and a deleterious missense variant in this case is consistent with the observed rapidly fatal neonatal course. Table 1 summarizes previously reported Japanese cases of CACT deficiency^11–13,19,20^.

**Table 1:** Clinical and genetic features of CACT deficiency cases in Japan.

Patient	Gender	Age at onset	Neonatal onset (+/−)	Outcome (alive/dead)	Age at last follow-up	Variant 1	Variant 2	Refs.
1	M	6 m	−	Alive	2 y	c.3G>A p.(Met1?)	IVS4+1g>t	Lopriore 2001^19^; Yamaguchi 2012^20^
2	M	2 d	+	Dead	2y9m	c.199-10t>g	c.576G>A p.(Trp192Ter)	Fukushima 2013^11^
3	M	2 d	+	Dead	3 d	c.106-2a>t	c.576G>A p.(Trp192Ter)	Fukushima 2013^11^
4	F	2 d	+	Alive	10y8m	c.824G>A p.(Arg275Gln)	c.824G>A p.(Arg275Gln)	Chinen 2020^12^
5	M	2 d	+	Dead	2y2m	c.824G>A p.(Arg275Gln)	c.824G>A p.(Arg275Gln)	Chinen 2020^12^
6	M	0 d	+	Alive	5y6m	c.824G>A p.(Arg275Gln)	c.824G>A p.(Arg275Gln)	Chinen 2020^12^
7	F	2 d	+	Alive	3y2m	c.824G>A p.(Arg275Gln)	c.824G>A p.(Arg275Gln)	Chinen (Japanese)^13^
8	F	1 d	+	Dead	2 d	c.334C>T p.(Gln112Ter)	c.824G>A p.(Arg275Gln)	Present case

Patients 5 and 6 are siblings.

*d* days, *F* female, *M* male, *m* months, *y* years.

From a clinical perspective, this case underscores the importance of considering inherited metabolic disorders in cases of sudden neonatal death, even in the absence of prior symptoms or abnormal screening results. Establishing a molecular diagnosis is essential not only for determining the cause of death but also for providing appropriate genetic counseling to the family. Identification of pathogenic variants allows carrier testing of parents and enables informed reproductive planning, including options such as prenatal diagnosis and preimplantation genetic testing.

In conclusion, we report a case of sudden neonatal death in which compound heterozygous *SLC25A20* variants were identified through post-mortem genetic analysis. Genetic findings, together with clinical and radiological features, supported the diagnosis of CACT deficiency. This case highlights the diagnostic value of post-mortem genetic analysis for early fatal metabolic disorders and contributes to expanding our understanding of CACT deficiency.

## HGV Database

The relevant data from this Data Report are hosted at the Human Genome Variation Database at https://doi.org/10.6084/m9.figshare.hgv.3663. https://doi.org/10.6084/m9.figshare.hgv.3666.
